# Supplementary material for: A Swedish Familial Genome-Wide Haplotype Analysis Identified Five Novel Breast Cancer Susceptibility Loci on 9p24.3, 11q22.3, 15q11.2, 16q24.1 and Xq21.31
Source: Int J Mol Sci. 2023 Feb 24;24(5):4468. doi: 10.3390/ijms24054468 (PMC10003706; doi:10.3390/ijms24054468)
Supplement: Supplementary file 1 [file ijms-24-04468-s001.zip › ijms-2126966-supplementary.pdf]

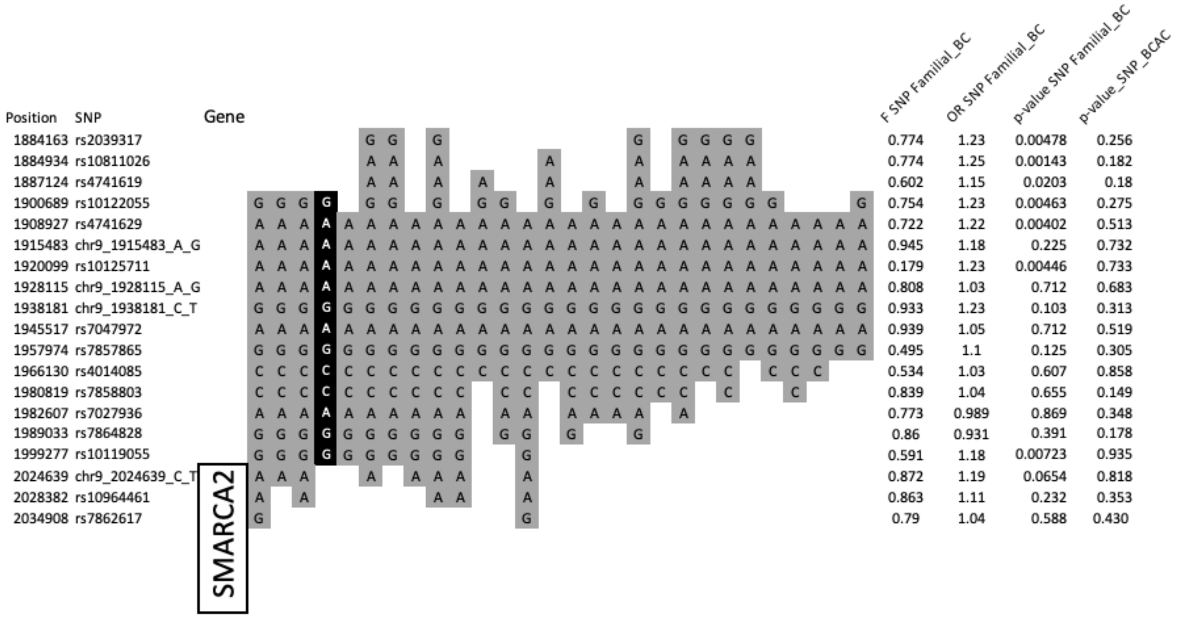

Figure S1 Locus 9p24.3, 28 significant haplotypes sorted by OR (Figure 1 in the article). The haplotype with the lowest p-value is marked in black and other significant haplotypes in gray. Additional individual SNP data to the right – first column = SNP frequency (F), second column = SNP OR, third column = SNP p-value. The fourth column to the right is comparable SNP p-value collected from BCAC homepage.

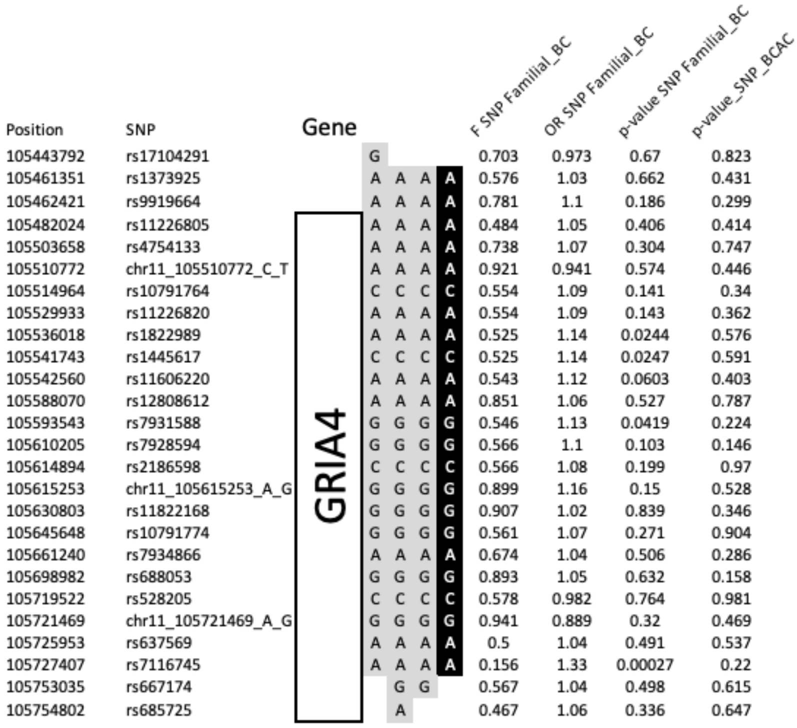

Figure S2 Locus 11q22.3, one significant haplotype and three borderline significant familial haplotypes sorted by OR (Figure 2 in the article). The significant haplotype is marked in black and borderline significant in light gray. Additional individual SNP data to the right – first column = SNP frequency (F), second column = SNP OR, third column = SNP p-value. The fourth column to the right is comparable SNP p-value collected from BCAC homepage.

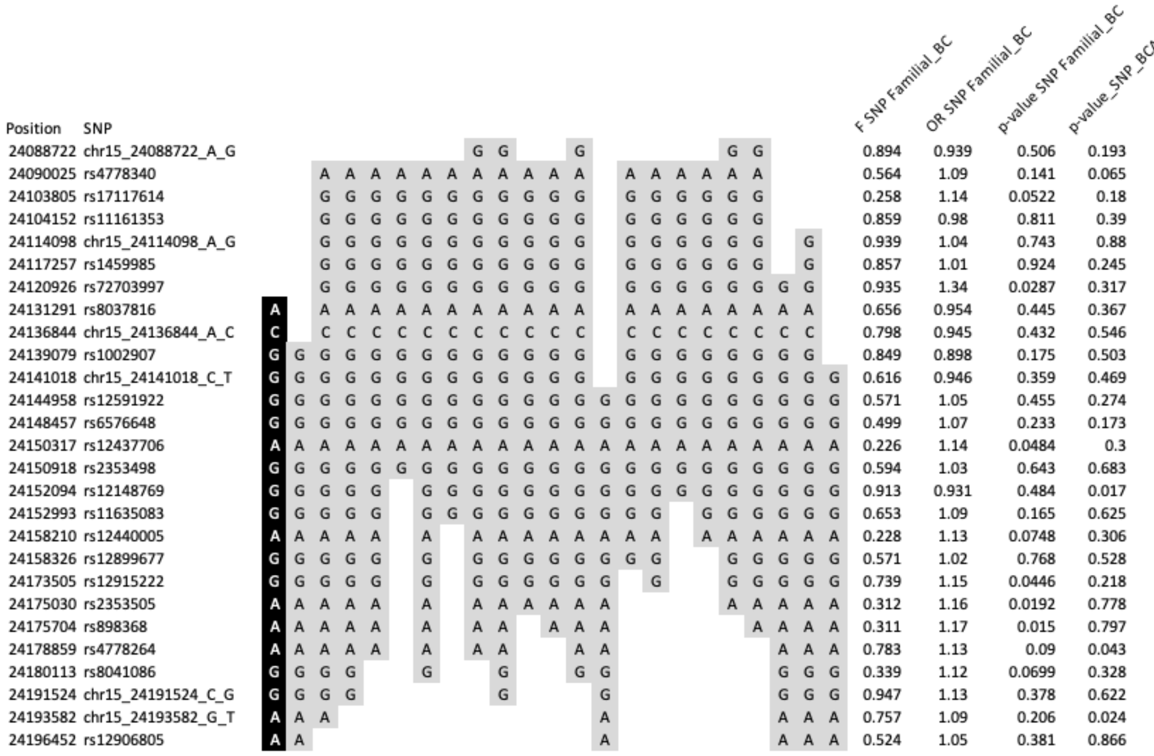

Figure S3 Locus 15q11.2, one significant and 22 borderline significant familial haplotypes sorted by OR (Figure 3 in the article). The significant haplotype is marked in black and borderline significant in light gray. Additional individual SNP data to the right – first column = SNP frequency (F), second column = SNP OR, third column = SNP p-value. The fourth column to the right is comparable SNP p-value collected from BCAC homepage.

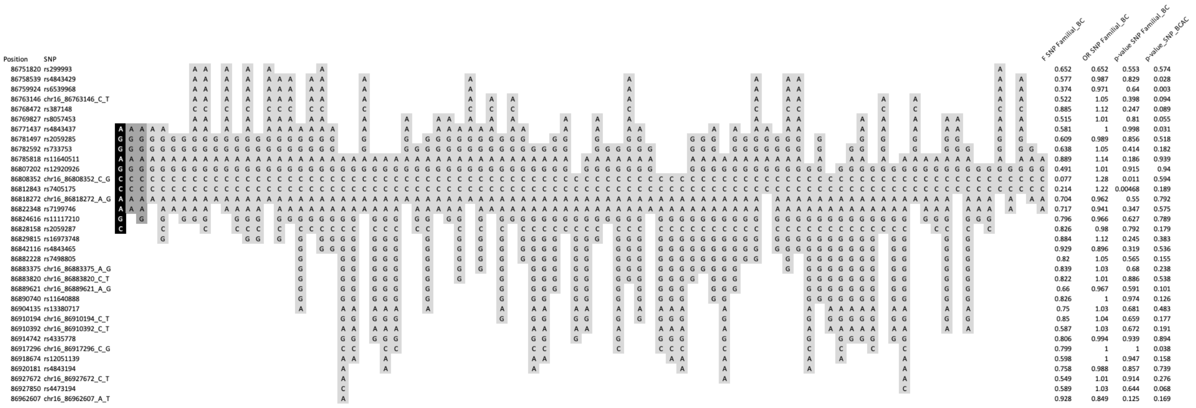

Figure S4 Locus 16q24.1, 3 significant and 85 borderline significant familial haplotypes sorted by OR (Figure 4 in the article). The haplotype with the lowest p-value is marked in black, other significant haplotypes in dark gray and borderline significant in light gray. Additional individual SNP data to the right – first column = SNP frequency (F), second column = SNP OR, third column = SNP p-value. The fourth column to the right is comparable SNP p-value collected from BCAC homepage.

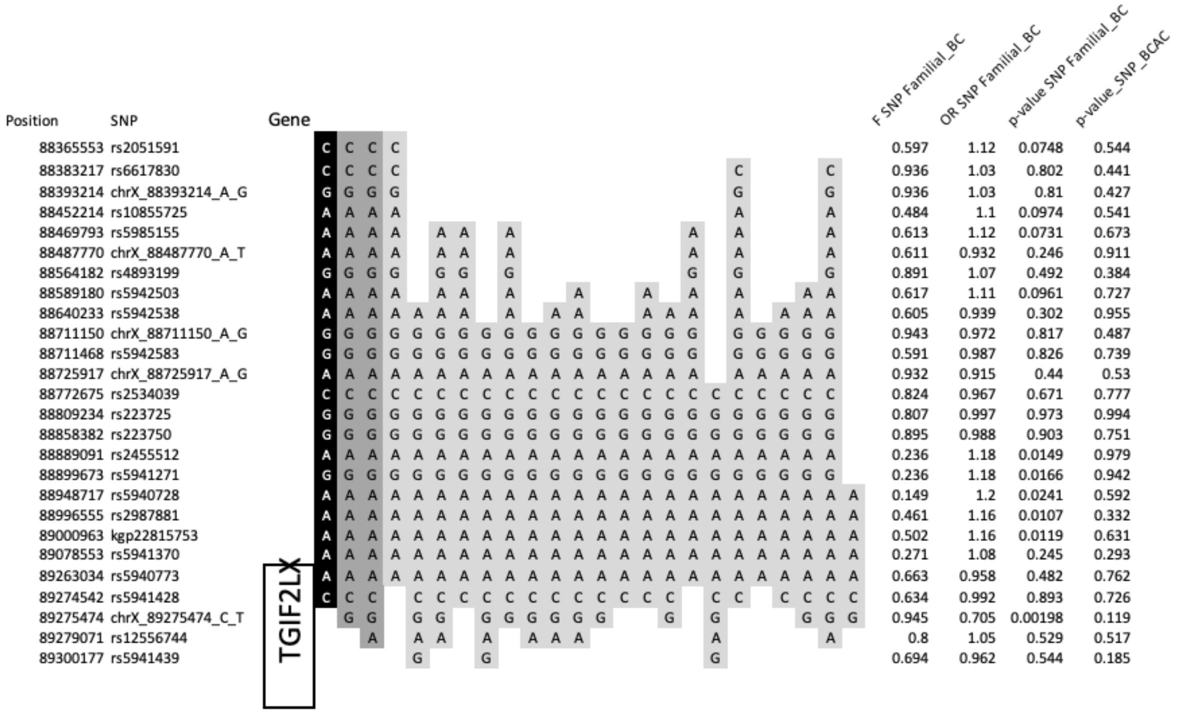

Figure S5 Locus Xq21.31, 3 significant and 21 borderline significant familial haplotypes sorted by OR **(**Figure 5 in the article). The haplotype with the lowest p-value is marked in black, other significant haplotypes in dark gray and borderline significant in light gray. Additional individual SNP data to the right – first column = SNP frequency (F), second column = SNP OR, third column = SNP p-value. The fourth column to the right is comparable SNP p-value collected from BCAC homepage.
